# Supplementary material for: Performance evaluation of the diaxxoPCR system for rapid and user-friendly stool-based diagnosis of trichuriasis, ascariasis and strongyloidiasis in Mozambique
Source: PLoS Negl Trop Dis. 2025 Nov 11;19(11):e0013711. doi: 10.1371/journal.pntd.0013711 (PMC12622830; doi:10.1371/journal.pntd.0013711)
Supplement: S1 File — (PDF) [file pntd.0013711.s002.pdf]

Fast PCR testing procedure
Quick Guide

1

Load **4.5 µL** of **extracted sample** into wells of diaxxoPod.

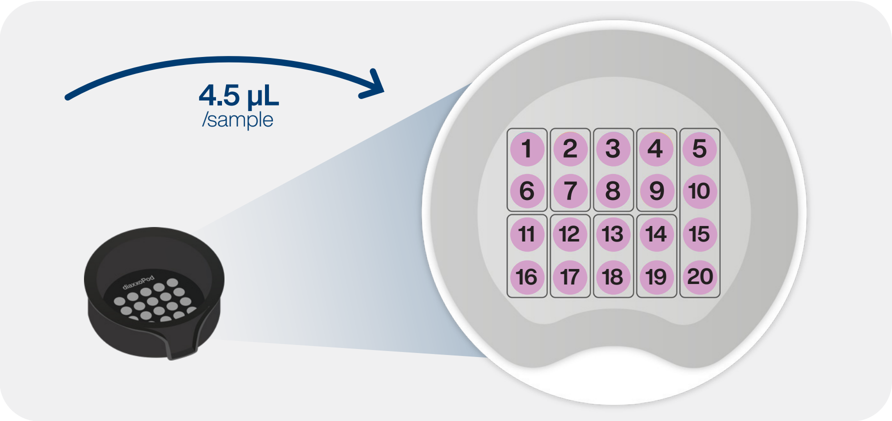

2

Load **1.7 mL** (2x 0.85 mL) of **covering liquid** onto diaxxoPod to seal the wells.

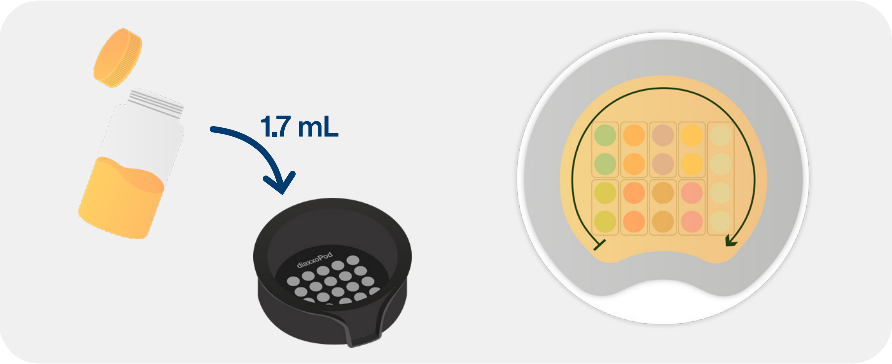

3

Open diaxxoPCR lid, **insert** diaxxoPod. Close the lid and press "**RUN EXPERIMENT"** to run the PCR test.

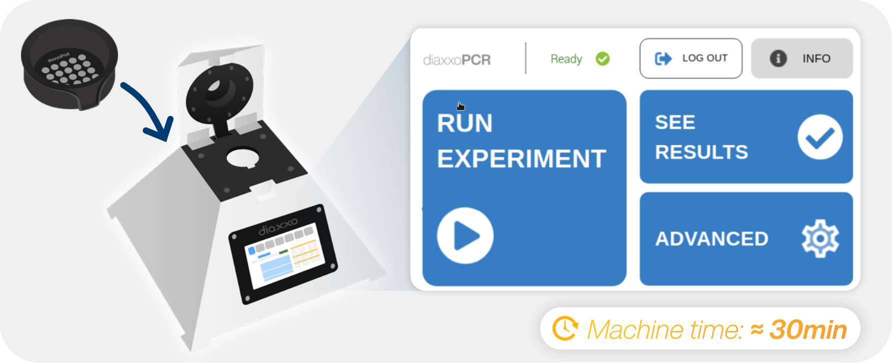

4

When the test is concluded, you can read the **test results** on the **screen**.

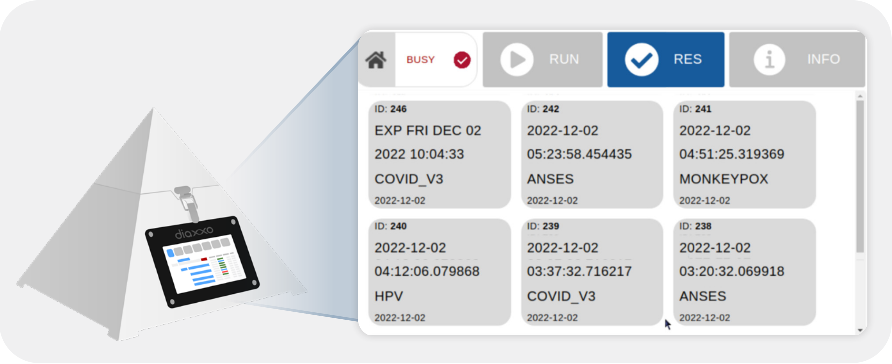

Disclaimer

Please consult the corresponding *Instructions For Use* for more details about materials, procedures and safety measures.
