# Supplementary material for: Performance evaluation of the diaxxoPCR system for rapid and user-friendly stool-based diagnosis of trichuriasis, ascariasis and strongyloidiasis in Mozambique
Source: PLoS Negl Trop Dis. 2025 Nov 11;19(11):e0013711. doi: 10.1371/journal.pntd.0013711 (PMC12622830; doi:10.1371/journal.pntd.0013711)
Supplement: Attachment — Submitted filename: Rebuttal_lettter_v02_25OCT2025_AMJ_MB_clean.docx [file pntd.0013711.s005.docx]

**Performance evaluation of the diaxxoPCR system for rapid and user-friendly stool-based diagnosis of trichuriasis, ascariasis and strongyloidiasis in Mozambique**

PNTD-D-25-00798

Messa Jr. *et al*.

Dear Drs Ionica and Chai:

Thank you all for your favourable preliminary appraisal on Ms. PNTD-D-25-00798, and for making available to us the Reviewers' comments, which we have considered carefully. Please see below a thorough point-by-point response to each issue raised by Reviewers #1 and #2. Changes introduced in the main body of the text have been highlighted in red for better identification.

**Reviewer´s #1 comments**

Methods:

1. Could authors please comment on the comparative PCR: is this an in-house developed or commercial assay? Is it fully-validated or accredited? Why was this test used as comparison?

*Response: The authors would like to clarify that for this study, no new extraction or qPCR protocols were developed. All the steps used to perform the DNA extraction and the multiplex qPCRs (ANAS and ST panels) have been previously described by Kaisar* et al*. 2017, (doi:10.1017/S0031182017000129). This was selected as the reference method for direct PCR vs PCR comparison in this study because it has been extensively validated and has produced consistent results when assessed through the international Helminth External Molecular Quality Assessment Scheme (HEMQAS) provided by the Dutch Foundation for Quality Assessment in Medical Laboratories (SKML). Although not currently available commercially as an assay, all the reagents and consumables can be purchased and it was adopted as the qPCR for testing samples in the ALIVE Clinical Trial, with the CISM Laboratory also participating in the HEMQAS.*

*For the diaxxoPCR for which we describe the performance in this study (compared to the multiplex qPCR), the only modification was the use of 6-Carboxyfluorescein (6-FAM) in the probes instead of the fluorophores described by Kaisar* et al*.; however, the oligonucleotide sequences and the respective concentrations were kept. The diaxxoPCR system itself is commercially available (with a CE certification); however, the custom cartridges/pods used in this study are not yet available for purchase, and were prepared for this study based on a specific order and agreement with the manufacturer.*

Results:

2. The data are well presented.

*Response: We thank Reviewer #1 for their assessment on this matter.*

Conclusions:

3. Line 40/41: “demonstrate the performance” in study C needs to be clarified further whether this is analytical performance.

*Response: The authors agree with Reviewer #1 and would like to clarify that Study C was performed to assess technical performance. This has been clarified in the text (Lines 40 and 191).*

4. Line 54/55: “low resource laboratory settings”. This needs to be justified – e.g. costing, ease of use, etc.

*Response: The authors would like to clarify that indeed we use the expression “low resource laboratory settings” to cover multiple dimensions, including in terms of cost (for equipment, consumables), availability of skilled personnel, complexity of procedures, ease of use and other requirements (e.g. cold-chain, infrastructure, reliable network of providers). Many of the settings facing these challenges are found in the Global South (Sub-Saharan Africa, Latin America and South Asia). We have highlighted where this is covered in the body of the manuscript and now indicate where we made slight modifications to reflect this comment from the Reviewer (lines: 55, 101-109, 507).*

Summary and general comments:

This is a very well-written study examining laboratory validation of a commercial cartridge-based PCR system on helminth detections. It has great relevance to the diagnostics accessibility in resource-limited settings.

*We thank Reviewer #1 for their preliminary positive appraisal of the merits of our study.*

I suggest the authors address the following points:

5. To discuss at the introduction the context of this study in the absence of true “gold standard” (10.1016/j.ijpara.2014.05.009)

*Response: We thank Reviewer #1 for the suggestion of including a statement that indicates there is currently no “gold standard” for STH diagnosis supported by Nikolay, Brooker and Pullan (2014). We opted to make this clarification in the discussion of the manuscript (lines 360-361).*

6. Discuss limitations of this study: e.g. monitoring of load or intensity of disease, evaluation of treatment response, shedding dynamics are not in scope

*Response: The authors agree with Reviewer #1 that the points raised are indeed limitations of the work we have presented in this manuscript and that they are beyond the scope of our study, since we are focusing on assessing the technical performance of diaxxoPCR compared directly to a “standard” multiplex qPCR. Although we used stool samples collected during a clinical trial where treatment response was one of the endpoints assessed, this evaluation is beyond the scope of the work currently presented. In this work, we have also performed a comparison between the Cqs (diaxxoPCR)* vs*. Cts (qPCR), which offer a quantitative measurement; however, it cannot be correlated directly to the intensity of infection, as has already been covered in lines 410-421.*

7. Line 69: “population-based surveys” have not been evaluated in this study, hence should not be included in the conclusion.

*Response: The authors thank Reviewer #1 for pointing this out; however, we respectfully disagree. Since the evaluation was performed using samples collected for the ALIVE trial (NCT05124691) in a setting representative of where observational studies and clinical trials are typically conducted, the conclusions of this evaluation can also be applied to population-based surveys.*

8. Suggest discuss the PPV and NPV in the context of disease prevalence in Mozambique.

*Response: The authors thank Reviewer #1 for the suggestion, however, we respectfully disagree. We are describing the findings of a technical comparison of diaxxoPCR and qPCR. We do not see the relevance of discussing the PPV and NPV in the context of prevalence of these STH in Mozambique which are beyond the scope of this technical evaluation.,*

9. Line 457-464: authors many want to consider rephrasing this as a general issue rather than a specific company issue, e.g. “a well-developed instrument support programme is required to ensure sustainable testing in Africa due to limited capability for instrument technical support by individual companies.”

*Response: The authors agree with Reviewer #1 that this is indeed an issue that affects not only the diaxxoPCR system but is generalizable to all qPCR systems/reagents providers. We have included a statement in the manuscript to make this clear (lines: 468-470).*

**Reviewer´s #2 comments**

We did not identify any comments or questions from Reviewer #2 in the letter shared with us by the journal; however, we appreciate their assessment of our manuscript.
